# A Non-Invasive IR Sensor Technique to Differentiate Parkinson’s Disease from Other Neurological Disorders Using Autonomic Dysfunction as Diagnostic Criterion

**DOI:** 10.3390/s22010266

**Published:** 2021-12-30

**Authors:** Brindha Anbalagan, Sunitha Karnam Anantha, Sridhar P. Arjunan, Venkatraman Balasubramanian, Menaka Murugesan, Kalpana R

**Affiliations:** 1 EIE Department, SRM Institute of Science and Technology, Kattankulathur 603203, India; brindhaa@srmist.edu.in; 2 Safety and Environmental Group, Department of Atomic Energy, IGCAR, Kalpakkam 603102, India; bvenkat@igcar.gov.in (V.B.); menaka@igcar.gov.in (M.M.); 3 Department of Neurology, SRM Medical College Hospital and Research Centre, Kattankulathur 603203, India; kalpanasenthilkumar@rediffmail.com

**Keywords:** thermography, cold stress test, autonomic dysfunction, vasoconstriction, essential tremor, Parkinson’s disease, skin temperature

## Abstract

Early diagnosis of Parkinson’s disease (PD) plays a critical role in effective disease management and delayed disease progression. This study reports a technique that could diagnose and differentiate PD from essential tremor (ET) in its earlier stage using a non-motor phenotype. Autonomic dysfunction, an early symptom in PD patients, is caused by α -synuclein pathogenesis in the central nervous system and can be diagnosed using skin vasomotor response to cold stimuli. In this study, the investigations were performed using data collected from 20 PD, 20 ET and 20 healthy subjects. Infrared thermography was used for the cold stress test to observe subjects’ hand temperature before and after cold stimuli. The results show that the recovery rate of hand temperature was significantly different between the groups. The data obtained in the cold stress test were verified using Pearson’s cross-correlation technique, which showed that few disease parameters like medication and motor rating score had an impact on the recovery rate of hand temperature in PD subjects. The characteristics of the three groups were compared and classified using the k-means clustering algorithm. The sensitivity and specificity of these techniques were analyzed using an Receiver Operating Characteristic (ROC) curve analyzer. These results show that this non-invasive technique can be used as an effective tool in the diagnosis and differentiation of PD in its early stage.

## 1. Introduction

There are one million people living with PD and 60,000 new cases are diagnosed every year, across the world [1]. Men are approximately 1.5 times more prone to PD than women, due to their lifestyle differences [2]. ET affects 4% of adults aged above 40 years. Due to its heterogeneous condition, 30–50% of ET cases are misdiagnosed [3]. Unlike PD, the diagnostic tool used to diagnose ET is the TETRAS rating scale [4]. Numerous advantages of early symptomatic therapeutic intervention in PD are evidenced in the medical literature [5]. The diagnosis of PD in its early stage and instituting suitable treatment will slow the liability of disease progression. Various non-motor and motor symptoms of PD are mentioned in Table 1. Some critical features of the non-motor phase are fatigue, cognitive impairment, depression and anxious mood, improper sleep, autonomic dysfunction, hyposmia, etc. With the onset of the motor phase, patients will start experiencing speech difficulties, drooling, chewing and swallowing difficulty, facial expression change, problems with handwriting, resting tremors (4–6 Hz), imbalanced walking, bradykinesia, freezing of legs while standing and a need for assistance with walking [6,7]. There is a lot of scope in diagnosing the disease early (stage 1) in order to improve the quality of living in PD subjects and this work has been done to achieve the early diagnosis of PD.

### Pathophysiology of PD

The loss of dopaminergic neurons in regions of the substantia nigra, striatal projections and brainstem regions leads to a neurological disorder, i.e., PD [8,9]. This loss of neurons leads to the loss of nerve fibers responsible for motor functions [10]. The pathogenesis underlying the small nerve fibers of peripheral neuropathy shows that an accumulation of pathologic (phosphorylated) α -synuclein is the foremost reason for neuron death, potentially explaining the peripheral denervation found in PD [11,12]. Whereas, in the case of ET it is a nervous system disorder caused due to miscommunication in the cerebellum and other parts of the brain [13]. Autonomic dysfunction in PD subjects leads to vasoconstriction [14]. In addition, a study proved that sympathetic dysfunction may be present in ET subjects [15]. In the early stages of PD, autonomic dysfunction affects the control of body temperature due to vasoconstriction. The hyper-reactive vasoconstriction in human blood vessels is known as Raynaud’s phenomenon. Due to prolonged vasoconstriction, subjects experience cold limb phenomena, often accompanied by pain [16,17]. Among the non-motor phenotypes of PD, autonomic dysfunction is an essential category. A literature survey showed that autonomic dysfunction could be a biomarker for the diagnosis of PD in the early stages [18].

## 2. Materials and Methods

**Figure 1:**
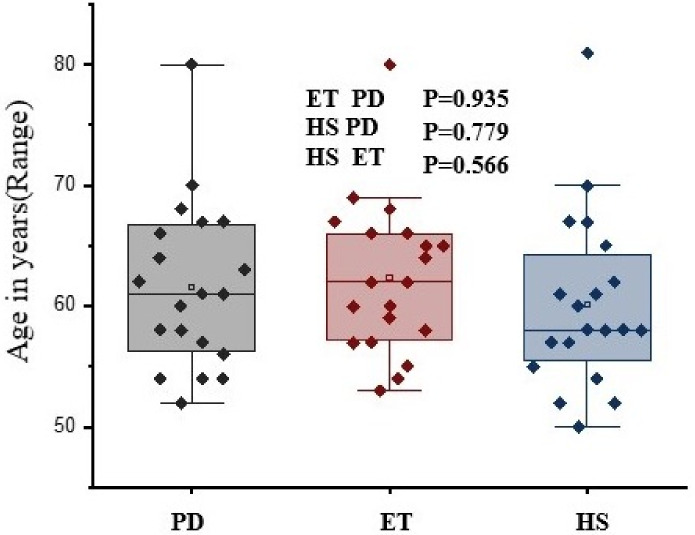
Comparison of mean age of the participants.

The current cross-sectional study involved 60 subjects. Table 2 shows the selection criteria for PD and ET subjects. Data collection was performed by explaining the procedure and with proper consent from patients. A neurologist from the SRM Medical College Hospital and Research Centre helped select the PD and ET subjects with sympathetic cardiac denervation (>6 months) for autonomic nervous dysfunction. There were specific exclusion criteria such as no current smoking habits, no infection or fever before two weeks of test, history of acute myocardial infarction, adrenal gland diseases, arterial disease that could affect skin temperature, or the presence of any other neurological disorders. The subjects were selected with similar baseline characteristics such as age, sex, BMI, etc., as shown in Figure 1.

### 2.1. Experimental Setup

**Figure 2:**
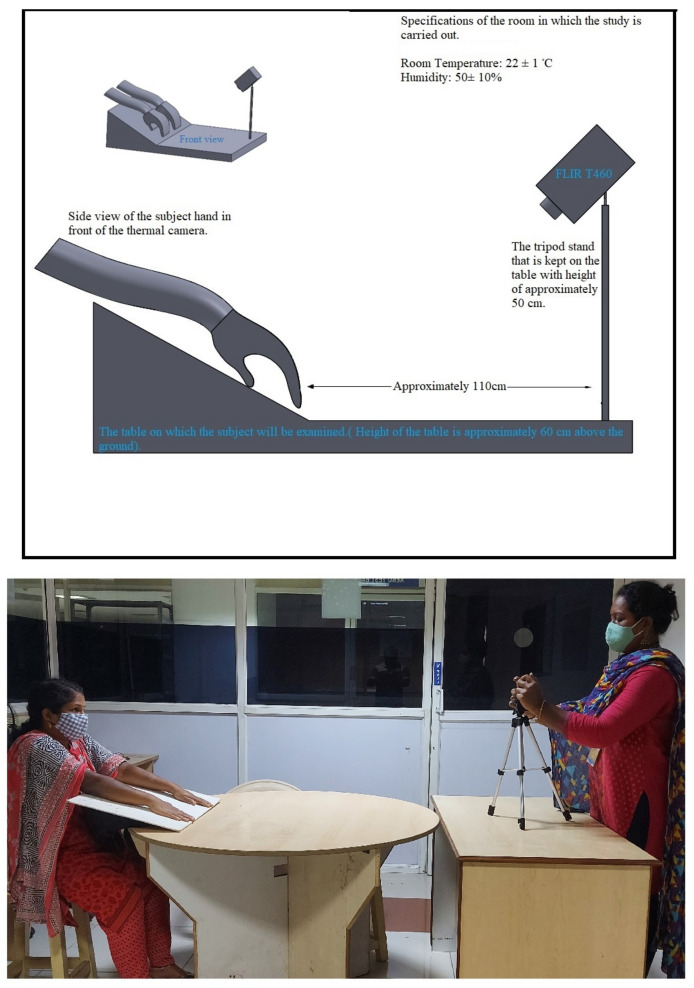
Experimental setup for the study.

The clinical setup, as explained in the reference [19], was followed. Clinical examination was performed in a controlled room of dimensions not less than 10 m ${}^{2}$ . The humidity of the room was maintained at 50 ± 10%, and the room temperature was held at 22 ± 1 ${}^{\circ }$ C. For the thermographic recording, the FLIR T460, 320x240 60 Hz Researcher infrared detector with a thermal sensitivity < 0.03 ${}^{\circ }$ C was used. The thermal camera was aligned in a hold-on position using a tripod stand adjusted to approximately 110 cm above the floor and 110 cm away from the subject’s hand as shown in Figure 2. The maximum and minimum temperatures of the test were 18 ± 1 ${}^{\circ }$ C and 40 ± 1 ${}^{\circ }$ C. The subjects who came for the test were rested for 15 min for adaptation of the skin temperature to room temperature. After explaining the detailed procedure to the subject and the attendee, written consent was obtained. The subject was also advised to convey if they felt any discomfort during the test. The subjects were made to sit in the clinical setup with their elbows on a table at a 60 ${}^{\circ }$ angle to the camera and their hands hanging down. Enough care was taken so that there was no contact of the hand with the table to avoid any deviation in skin temperature. Before performing the cold stress test, the baseline temperature (reference temperature) of the dorsum was acquired. After this, a bowl full of cold water at 3 ± 1 ${}^{\circ }$ C was placed for the patient to immerse both hands to the wrist wrapped in a latex glove. The guidelines of medicine were followed [20]. After two minutes post-immersion, the glove was carefully removed from the hand without touching the skin. Immediately post-immersion, the decrease in skin temperature was measured continuously from 0 to 6 min.

### 2.2. Data Collection and Statistical Analysis

**Figure 3:**
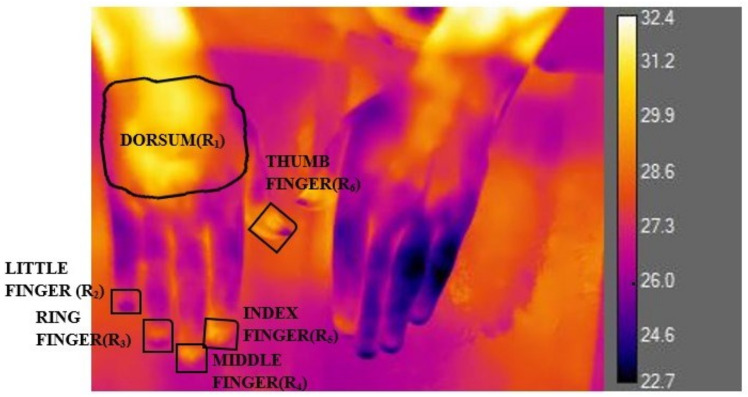
Finding thermal gradients in 6 ROIs to find the DDD.

Origin and IBM SPSS statistics tools were used for data analysis. To calculate the hand temperature from the thermal image, six regions of interest were described [21]. The temperature of the dorsum of the wrist was manipulated by marking a square ROI of cm ${}^{2}$ and 5 ROIs in the distal phalanx of 5 fingers whose areas were 1.75 cm ${}^{2}$ , as shown in in Figure 3.

The dorsal-distal difference (DDD) of each finger with the dorsum of the wrist was calculated. DDD was basically the difference between the temperature of the dorsum and the distal of each finger. Among the 5 ROIs in the distal region, the third finger showed a significant change in DDD. Thus, the DDD of the third finger was preferred for further analysis.The recovery rate (RR) of each hand after 6 min was calculated to diagnose vasoconstriction in the subject. The RR was calculated as shown in Equation (1) [22]:

(1) $$\begin{array}{c} \mathrm{RR}=\frac{T_{6post-immersion}-T_{0post-immersion}}{T_{0pre-immersion}-T_{0post-immersion}}\times 100\% \end{array}$$

where

T ${}_{0pre-immersion}$ = baseline skin temperature before CST,

T ${}_{0post-immersion}$ = skin temperature immediately after CST,

T ${}_{6post-immersion}$ = skin temperature at 6 min post-CST.

In this study, three groups were investigated. There were many quantitative and categorical data values considered for all 3 groups. Quantitative data are represented as mean ± SD and a non-parametric Mann–Whitney test was used to analyze the data. Categorical data are represented as median (range) and a chi-squared test was used to assess the association among groups. Multiple linear regression analysis was used to understand the association of (categorical and quantitative) independent and dependent data. The one-way ANOVA with Tukey’s test for multiple comparisons, was used to obtain the significance between means of categorical data and quantitative data. The k-means clustering algorithm was then used to group the observed data into PD and non-PD. Sensitivity and specificity of the test to differentiate between PD and non-PD subjects was measured using a receiver operating characteristic (ROC) curve with non-parametric conditions.

## 3. Results

**Figure 4:**
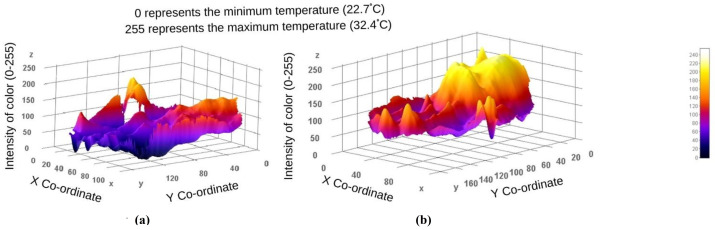
The 3D surface plot for the hand thermography of (**a**) tremor-affected and (**b**) normal hand of PD patient.

Table 2 shows the characteristics of PD and ET subjects that include both categorical and quantitative variables (H&Y score, UPDRS-ADL score, cardiac ( ${}^{123}$ I) MIBG scintigraphy, necessary drugs (mg)). The subjects were selected in such a way that PD subjects’ age groups were not significantly different when compared to ET (61.6 ± 6.81 vs. 62.35 ± 6.33; *p* = 0.935) and healthy subjects (61.6 ± 6.81 vs. 60.15 ± 7.22; *p* = 0.779). However, the mean ages of ET and healthy subjects were not significantly different (62.350 ± 6.33 vs. 60.15 ± 7.22; *p* = 0.566). The BMIs of the three groups were compared, and no significant difference was observed (25.46 ± 2.77 vs. 23.813 ± 2.37 vs. 25.79 ± 2.76; *p* = 0.047). There was no significant difference in the heart rate, hypertension and subjective hyposmia between the groups and within the groups. Most of the PD subjects had subjective hyposmia (45%, 30% and 10%; *p* < 0.0001). For the patients with diabetes mellitus, heart rate was not significantly different between the groups. Thus, the selected groups were homogeneously distributed. The thermal distribution in a tremor-affected hand and a normal hand of a PD patient was plotted as shown in Figure 4. The value 0 or black color shown in the scale represents the minimum temperature in the field of view (i.e., in this figure the minimum temperature was 22.7 ${}^{\circ }$ C and the corresponding color is black) and 255/white represents the maximum temperature (i.e., 32.4 ${}^{\circ }$ C). This plot shows the temperature distribution in the selected ROI. Thus, PD subjects showed greater thermal asymmetry between the hands in the 3D surface plot.

**Figure 5:**
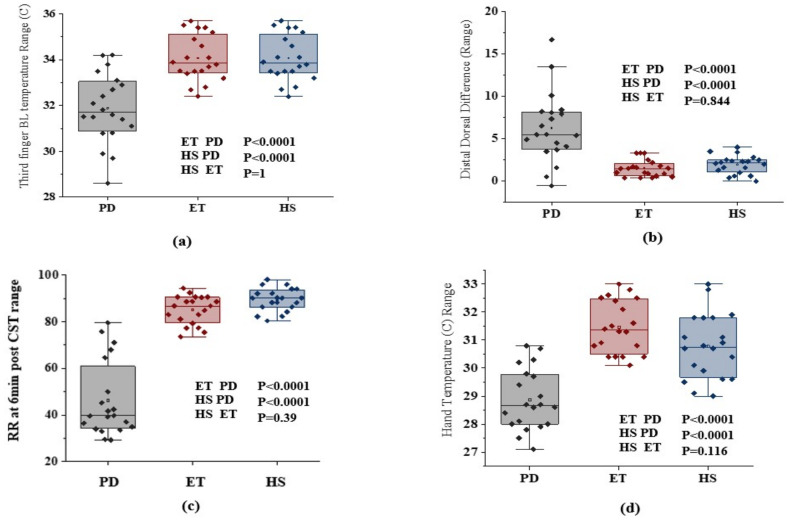
Comparison of three groups: (**a**) distribution of third finger BL temperature ©; (**b**) distribution of DDD of third finger; (**c**) distribution of RR at 6 min post-CST ©; (**d**) distribution of hand temperature ©.

The baseline thermographic analysis within the groups and between the groups was calculated using ANOVA. The characteristic thermal gradient in the six ROIs for three groups showed a significant population variance [(F(17,342) = 27.47 (*p* < 0.0001)]. The cold stress test was performed in both hands for all three groups of subjects. In this study, PD patients evidenced a low mean hand temperature when compared to ET (*p* < 0.0001) and HS (*p* < 0.0001). The thermograph was recorded for 0 to 6 min continuously for both hands. The thermographic study was performed for both the hands in all three groups of subjects. Hands that showed delayed temperature recovery were compared with each other and a lower RR after 6 min was observed in PD subjects (46.24 ± 16.13%, 85.18 ± 6.09% vs. 89.50 ± 4.89%; *p* < 0.0001). There was a significant difference between groups in recovery time (5.4 ± 0.6324, 8 ± 0.926 vs. 11.8 ± 1.473; *p* < 0.0001). Before the cold stress test, the baseline or normal temperature of PD showed a significant difference with ET and HS subjects (*p* < 0.0001) as shown in Figure 5.

**Figure 6:**
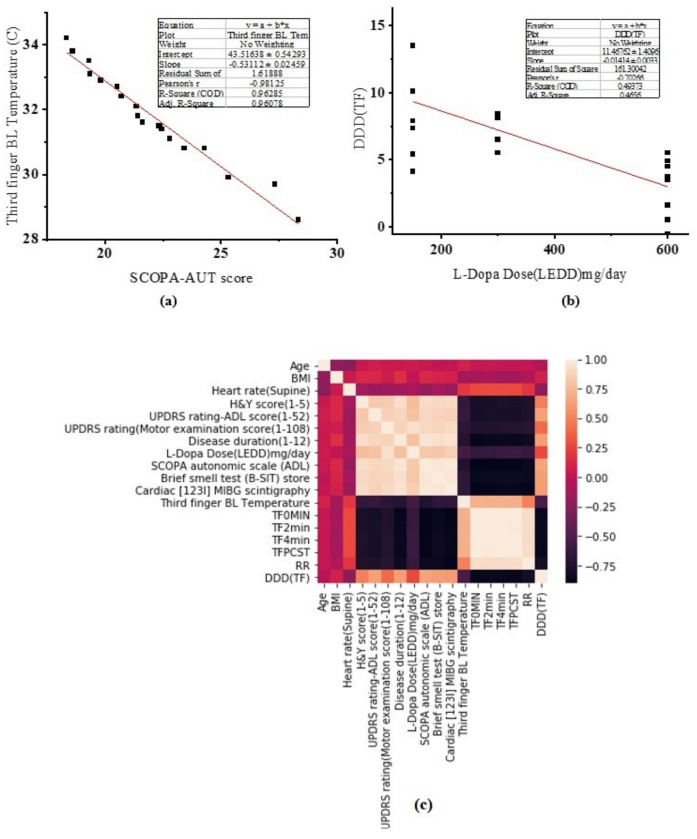
(**a**) Correlation of third finger baseline temperature with SCOPA-AUT score; (**b**) correlation of DDD with LEDD mg; (**c**) correlation of various parameters is shown in the heatmap.

To verify the data obtained in the cold stress test, Pearson’s cross-correlation technique was performed. The linear fit and regression analysis on the SCOPA autonomic scale with third finger BL temperature and DDD of the third finger with LEDD mg are shown in Figure 6a,b. The characteristics of PD subjects, such as H&Y score, UPDRS-ADL score, SCOPA-AUT score, disease duration, B-SIT, and cardiac ( ${}^{123}$ I) MIBG scintigraphy, were correlated with the recovery rate as shown in Figure 6c. In this test, age, BMI, heart rate, subjective hyposmia, arterial hypertension and diabetes mellitus did not correlate with recovery rate (*p* > 0.05). The correlation ‘r’ of recovery rate with B-SIT was −0.845 (*p* = 0.0005); with SCOPA-AUT score was −0.854 (*p* = −0.0003); with disease duration was −0.819 (*p* = 0.0005); with ADL score was −0.755 (*p* = 0.0003) and with H&Y score was −0.748 (*p* = 0.001). This correlation analysis showed that B-SIT, SCOPA-AUT, disease duration, ADL score and H&Y score impacts the recovery rate of hand temperature in PD subjects.

**Figure 7:**
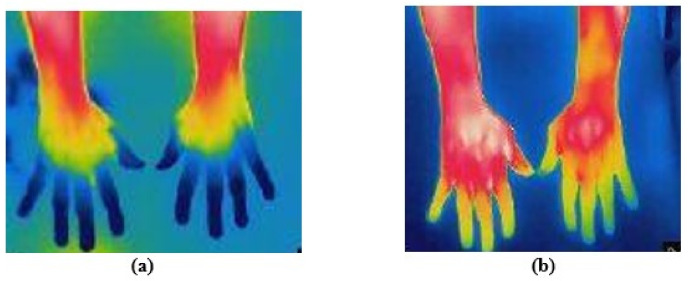
CST in a PD subject (**a**) 0 min after CST; (**b**) 6 min after CST.

**Figure 8:**
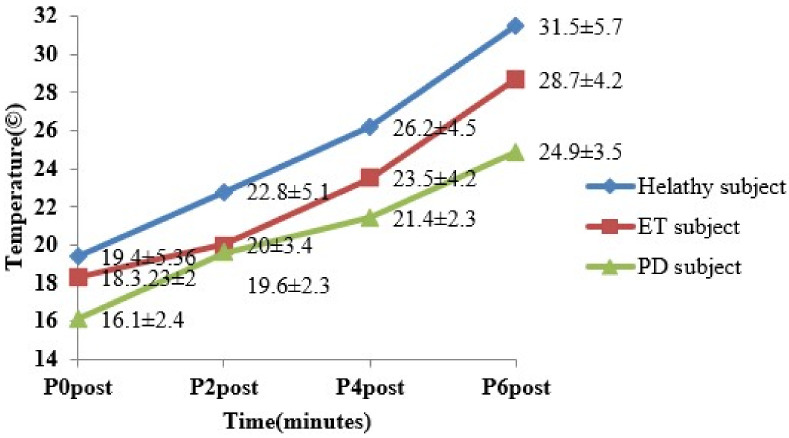
Comparison of cold stress test in healthy, ET and PD subjects.

It was also observed that the recovery rate of PD subjects was 32% less when compared to healthy and 25% less compared to ET subjects. The temperature recovery of the three groups after the cold stress test is plotted in Figure 7 and Figure 8.

**Figure 9:**
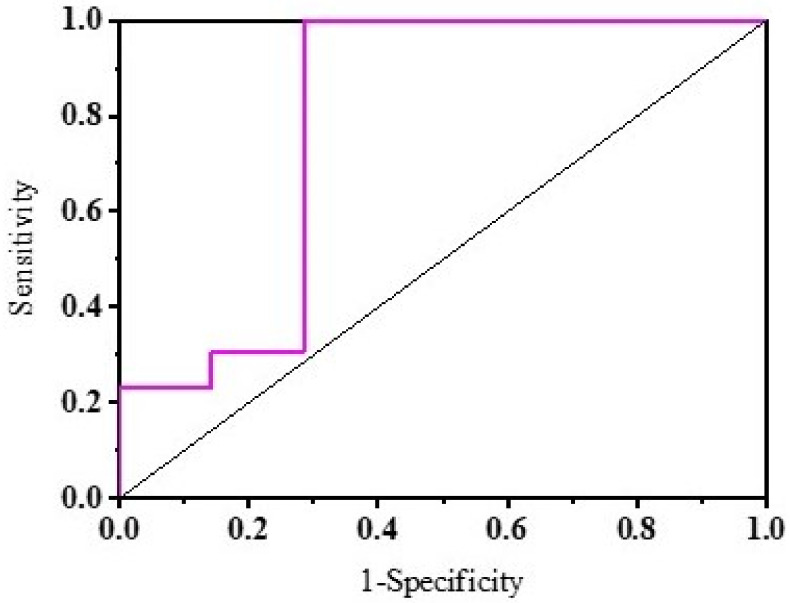
ROC curve for k-means clustering with RR as dependent variable.

PD subjects evidenced a lower thermal decrease when compared to ET and HS subjects and thus showed a lower RR and high DDD. Based on the recovery rate at 6 min the groups were classified into PD and non-PD. The ROC curve for this prediction was plotted as shown in Figure 9 and this parameter served to differentiate between PD and non-PD (AUC = 0.79; 95%CI 0.586–0.996) with the highest sensitivity = 0.923 and specificity = 0.715 at ${\mathrm{RR}}_{cutoff}$ = 47.65%.

## 4. Discussion

Traces of α -synuclein in PD subjects cause vasoconstriction (skin vasomotor dysfunction). Vasoconstriction causes reduced blood flow in blood vessels, which causes cold limbs in PD subjects. Vasoconstriction in PD subjects is caused due to autonomic dysfunction. A study on PD subjects found that 47% of them had autonomic dysfunction [23,24]. Autonomic dysfunction is much observed in subjects with neurodegenerative diseases such as Parkinson’s disease, Alzheimer’s disease and multiple sclerosis. Essential tremor is not generally characterized as a neurodegenerative disease, but in recent days it has been described so [23,25,26]. The SCOPA-AUT test was performed on a group of PD, ET and healthy subjects to observe the SCOPA-AUT score. This study showed that the PD patients had a significantly higher SCOPA-AUT score when compared to healthy subjects, whereas the SCOPA-AUT score was not significant between ET and healthy subjects. Thus, this study showed that PD subjects will show skin vasomotor reflex response to a cold stimulus [27,28].

A study involving 31 ET and 26 controls performed sympathetic skin response (SSR) and RR interval variation (RRIV) tests. This study was carried out to determine the health of the autonomic nervous system in ET. The mean amplitude of SSR was found to be significantly lower in ET subjects when compared to healthy subjects (*p* = 0.001). Based on the results obtained, it was concluded that ET subjects may have sympathetic dysfunction [15]. In another study, a non-invasive cold stress test proved to be an accurate and simple technique to find sympathetic skin dysfunction in PD subjects. This study involved 15 PD and 20 control subjects. Various PD characteristics were considered to observe the disease’s impact on SVR. The study was carried out in both hands, with one immersed in cold water (IH) and the other not immersed in cold water (NIH) regardless of motor laterality. The results showed that PD subjects had a lower mean baseline hand temperature (*p* = 0.037), and the PD subjects did not offer a regular cooling pattern compared to controls [19]. To find the α -synuclein deposits, peripheral autonomic dysfunction was evaluated in 20 PD subjects and compared with 19 healthy subjects. The study observed the temperature in hands, arms and legs during CST. The RR difference between PD and healthy subjects was observed to be significant in the 5^th^ distal phalanx [29]. Thus it has already been proved that thermal imaging can be used as a diagnostic tool for autonomic dysfunction. In the early stages of PD some patients are diagnosed to have ET and it has also been proved that PD patients will have autonomic dysfunction in the early stages. However, a comparative study of ET and PD subjects on autonomic dysfunction has not been performed, which is the novelty of this research work. In addition, this research work could bridge the challenges identified in previous research work.

## 5. Conclusions

In this study, it is evident from the analysis that the hand thermography of a PD subject varies between ET and healthy subjects due to CST. The thermal reduction is lower, and the recovery rate is slow after the CST in PD subjects and is due to vasomotor dysfunction. A reduced mean baseline temperature was observed in PD subjects when compared to ET and healthy subjects. Pain during CST was reported less frequently by PD patients than by ET and HS subjects (19% vs. 72%; *p* = 0.008). Thus, skin thermography and the CST technique proved to be effective non-invasive tests in the initial stages of PD. In order to further improve the temperature difference in a tremor-affected hand and a normal hand, the normalized heat-map of the hand can be designed in future studies. In addition to other evaluated symptoms in the UPDRS rating, CST will help diagnose the disease accurately and earlier to support the neurologist in developing suitable treatments. The results of this study have to be evaluated using a larger cohort of subjects so that these non-invasive tools can be used to detect PD in its early stage, which will help improve patients’ lifestyle and delay PD disease progression.

## Figures and Tables

**Table 1:** Various stages and symptoms of PD subjects.

Stages	Symptoms
Stage 0	Anxiety, mood disorders, dysautonomia, REM sleep disorders, sensory dysfunction.
Stage 1	Resting tremor symptoms on one side of the body, mild postural change, difference in walking patterns and difference in facial expressions occur.
Stage 2	Signs and symptoms in stage 1 affect both sides of the body; inability to perform tasks like dressing, bathing, eating, etc.; walking and gait problems.
Stage 3	Loss of balance, slowness of movements, frequent falls common.
Stage 4	Symptoms become severe and limiting. PD patients need assistance to stand, e.g., a walker to move.
Stage 5	Stiff legs, thus difficulty standing or walking. Mostly use walker.

**Table 2:** Baseline characteristics of Parkinson’s disease subjects, essential tremor and healthy subjects.

	PD Subjects (n = 20)	ET Subjects (n = 20)	Healthy Subjects (n = 20)	*p* Value
Age (years): mean ± SD (range)	61.6 ± 6.82	62.35 ± 6.34	60.15 ± 7.28	*p* = 0.585
Sex: (M/F)	13/7	12/8	12/8	*p* = 0.307
BMI (kg/m ${}^{2}$ ): mean ± SD (range)	25.46 ± 2.78	23.81 ± 2.38	25.8 ± 2.76	*p* = 0.470
Heart rate: mean ± SD (range)	66.7 ± 4.76	69.15 ± 5.86	68.15 ± 5.15	*p* = 0.343
Arterial hypertension (yes/no)	3/17	5/15	8/12	*p* < 0.0001
Subjective hyposmia (yes/no)	9/11	6/14	2/18	*p* < 0.0001
Diabetes mellitus (yes/no)	9/11	10/10	11/9	*p* = 0.159
Orthostatic hypotension (yes/no)	6/14	4/16	4/16	*p* = 0.211
B-SIT: mean ± SD (range)	6.98 ± 1.23	-	-	-
Cardiac ( ${}^{123}$ I) MIBG scintigraphy: mean ± SD	1.29 ± 0.12	-	-	-
SCOPA-AUT score: mean ± SD (range)	21.91 ± 2.8	5.02 ± 11.8	-	-
Disease duration: mean ± SD (range)	7.7 ± 1.95	7.3 ± 1.84	-	-
HY score: median (range)	(1–3) 2	-	-	-
UPDRS-ADL score (1–52): median (range)	(11–42) 21.5	-	-	-
UPDRS-Motor examination score (1–108): median (range)	(11–83) 54	-	-	-
Parkinsonian phenotype tremoric/akinetic-rigid/mixed	12/3/5	-	-	-
L-Dopa dose (LEDD) mg/day: median (range)	300 (150–600)	-	-	-
TETRAS rating score (0–4): median (range)	-	2 (1–3)	-	-
Propranolol with any other necessary drugs (mg): median (range)	-	350 (120–600)	-	-

## Data Availability

The data presented in this study are available on request from the corresponding author. The data are not publicly available since the data also include other clinical information about the patients.

## References

[1] Ball N., Teo W.P., Chandra S., Chapman J. (2019). Parkinson’s disease and the environment. Front. Neurol..

[2] Wong S.L., Gilmour H.L., Ramage-Morin P.L. (2014). Parkinson’s Disease: Prevalence, Diagnosis and Impact.

[3] Zesiewicz T.A., Chari A., Jahan I., Miller A.M., Sullivan K.L. (2010). Overview of essential tremor. Neuropsychiatr. Dis. Treat..

[4] Elble R.J. (2016). The essential tremor rating assessment scale. J. Neurol. Neuromed..

[5] Tinelli M., Kanavos P., Grimaccia F. (2016). The Value of Early Diagnosis and Treatment in Parkinson’s Disease: A Literature Review of the Potential Clinical and Socioeconomic Impact of Targeting Unmet Needs in Parkinson’s Disease.

[6] Recchia A., Rota D., Debetto P., Peroni D., Guidolin D., Negro A., Skaper S.D., Giusti P. (2008). Generation of a *α*-synuclein-based rat model of Parkinson’s disease. Neurobiol. Dis..

[7] Lee V.M.Y., Trojanowski J.Q. (2006). Mechanisms of Parkinson’s disease linked to pathological *α*-synuclein: New targets for drug discovery. Neuron.

[8] Tanner C. (1992). Occupational and environmental causes of parkinsonism. Occup. Med..

[9] Chinta S.J., Andersen J.K. (2005). Dopaminergic neurons. Int. J. Biochem. Cell Biol..

[10] Dawson T.M., Dawson V.L. (2003). Molecular pathways of neurodegeneration in Parkinson’s disease. Science.

[11] Nolano M., Provitera V., Estraneo A., Selim M.M., Caporaso G., Stancanelli A., Saltalamacchia A.M., Lanzillo B., Santoro L. (2008). Sensory deficit in Parkinson’s disease: Evidence of a cutaneous denervation. Brain.

[12] Volpicelli-Daley L.A., Luk K.C., Patel T.P., Tanik S.A., Riddle D.M., Stieber A., Meaney D.F., Trojanowski J.Q., Lee V.M.Y. (2011). Exogenous *α*-synuclein fibrils induce Lewy body pathology leading to synaptic dysfunction and neuron death. Neuron.

[13] Thenganatt M.A., Louis E.D. (2012). Distinguishing essential tremor from Parkinson’s disease: Bedside tests and laboratory evaluations. Expert Rev. Neurother..

[14] Berg D., Postuma R.B., Adler C.H., Bloem B.R., Chan P., Dubois B., Gasser T., Goetz C.G., Halliday G., Joseph L. (2015). MDS research criteria for prodromal Parkinson’s disease. Mov. Disord..

[15] Habipoglu Y., Alpua M., Bilkay C., Turkel Y., Dag E. (2017). Autonomic dysfunction in patients with essential tremor. Neurol. Sci..

[16] Pauling J.D., Flower V., Shipley J., Harris N.D., McHugh N.J. (2011). Influence of the cold challenge on the discriminatory capacity of the digital distal–dorsal difference in the thermographic assessment of Raynaud’s phenomenon. Microvasc. Res..

[17] Wigley F.M., Flavahan N.A. (1996). Raynaud’s phenomenon. Rheum. Dis. Clin. N. Am..

[18] Goldstein D.S., Sewell L., Sharabi Y. (2011). Autonomic dysfunction in PD: A window to early detection?. J. Neurol. Sci..

[19] Antonio-Rubio I., Madrid-Navarro C., Salazar-López E., Pérez-Navarro M., Sáez-Zea C., Gómez-Milán E., Mínguez-Castellanos A., Escamilla-Sevilla F. (2015). Abnormal thermography in Parkinson’s disease. Park. Relat. Disord..

[20] Ring E., Ammer K. (2012). Infrared thermal imaging in medicine. Physiol. Meas..

[21] Greenstein D., Gupta N., Martin P., Walker D., Kester R. (1995). Impaired thermoregulation in Raynaud’s phenomenon. Angiology.

[22] Akaogi Y., Asahina M., Yamanaka Y., Koyama Y., Hattori T. (2009). Sudomotor, skin vasomotor, and cardiovascular reflexes in 3 clinical forms of Lewy body disease. Neurology.

[23] Louis E.D., Faust P.L., Vonsattel J.P.G., Honig L.S., Rajput A., Robinson C.A., Rajput A., Pahwa R., Lyons K.E., Ross G.W. (2007). Neuropathological changes in essential tremor: 33 cases compared with 21 controls. Brain.

[24] Chandran V., Pal P.K. (2012). Essential tremor: Beyond the motor features. Park. Relat. Disord..

[25] Benito-León J., Bermejo-Pareja F., Louis E.D. (2005). Incidence of essential tremor in three elderly populations of central Spain. Neurology.

[26] Benito-Leon J. (2014). Essential tremor: A neurodegenerative disease?. Tremor Other Hyperkinetic Movements.

[27] Lee S.M., Kim M., Lee H.M., Kwon K.Y., Koh S.B. (2015). Nonmotor symptoms in essential tremor: Comparison with Parkinson’s disease and normal control. J. Neurol. Sci..

[28] Damian A., Adler C.H., Hentz J.G., Shill H.A., Caviness J.N., Sabbagh M.N., Evidente V.G., Beach T.G., Driver-Dunckley E. (2012). Autonomic function, as self-reported on the SCOPA-autonomic questionnaire, is normal in essential tremor but not in Parkinson’s disease. Park. Relat. Disord..

[29] Purup M.M., Knudsen K., Karlsson P., Terkelsen A.J., Borghammer P. (2020). Skin Temperature in Parkinson’s Disease Measured by Infrared Thermography. Parkinson Dis..

